# Successful Treatment of Genitofemoral Neuralgia Using Ultrasound Guided Injection: A Case Report and Short Review of Literature

**DOI:** 10.1155/2014/371703

**Published:** 2014-04-06

**Authors:** Harsha Shanthanna

**Affiliations:** St. Joseph's Hospital, Department of Anesthesiology, McMaster University, Health Sciences Centre 2U1, 1200 Main Street West, Hamilton, ON, Canada L8N 3Z5

## Abstract

A young male patient developed chronic, severe, and disabling right sided groin pain following resection of his left testicular cancer. Since there is considerable overlap, ultrasound guided, selective diagnostic nerve blocks were done for ilioinguinal, iliohypogastric, and genitofemoral nerves, to determine the involved nerve territory. It was revealed that genitofemoral neuralgia was the likely cause. As a therapeutic procedure, it was injected with local anesthetic and steroid using ultrasound guidance. The initial injection led to pain relief of 3 months. Subsequent blocks reinforced the existing analgesia and were sufficient to allow for maintenance with the use of analgesic medications. This case report describes the successful use of diagnostic selective nerve blocks for the assessment of groin pain, subsequent to which an ultrasound guided therapeutic injection of genitofemoral nerve led to long term pain relief. As a therapeutic procedure, genitofemoral nerve block is done in patients with genitofemoral neuralgia. Ultrasound allows for controlled administration and greatly enhances the technical ability to perform precise localization and injection. There are very few case reports of such a treatment in the published literature. Apart from the case report, we also highlight the relevant anatomy and a brief review of genitofemoral neuralgia and its treatment.

## 1. Introduction 

**Figure 1:**
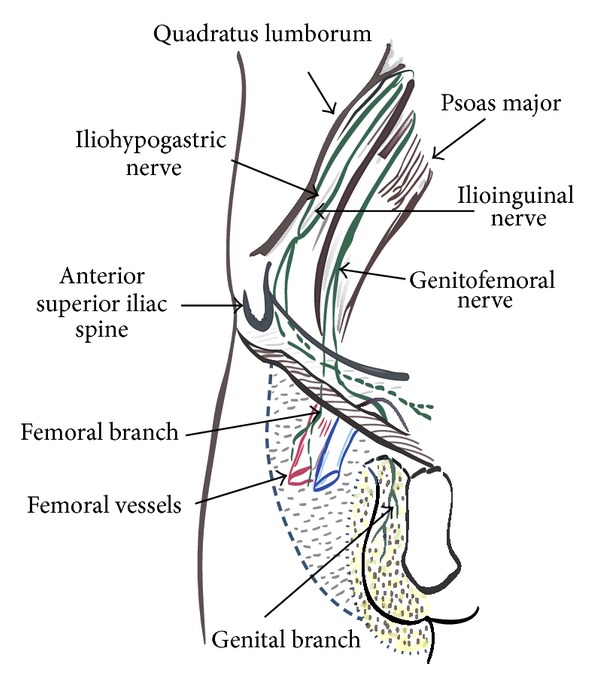
Anatomy of nerves around the inguinal region.

In 1942, Magee described the condition of pain and paresthesias in the distribution of genitofemoral nerve [1]. He called it as genitofemoral neuralgia (GFN), as it was not widely recognized. It was then reported to be commonly associated with appendicular surgeries. As shown in Figure 1, there is considerable overlap in the areas supplied from inguinal (IL), iliohypogastric (IH), genitofemoral (GF), and lateral cutaneous femoral nerve (LCFN) [2]. These nerves are quiet susceptible to injury following many lower abdominal and pelvic surgeries. Beneš et al. suggest using the term abdominoinguinal pain syndrome, as a common entity [3]. The diagnosis and treatment of these conditions are difficult and challenging. Although the exact incidence of GFN is not known, the incidence of chronic pain after inguinal hernia surgery is quoted around 12%–20% [4]. The inability to distinguish a GFN from ilioinguinal nerve pain can lead to unnecessary nerve exploration surgery and more morbidity [5]. Some have resorted to paravertebral nerve blocks at L1-L2 to alleviate the pain by blocking the common segmental origins [5]. In the following report we describe a patient of severe groin pain who underwent ultrasound guided selective nerve blocks, before being diagnosed and treated as GFN. This report highlights 2 important aspects. Firstly, it reports the unique advantage obtained from ultrasound in performing selective diagnostic nerve blocks around the groin. Secondly, it reports the successful management of GFN using ultrasound guided nerve block. Written permission has been obtained from the patient for this paper.

## 2. Case Description 

A 27-year-old male patient was referred to our pain clinic after having had orchidectomy for a left sided testicular cancer, 2 years earlier. He continued to have a persistent, severe pain in his right groin and scrotal area. The pain was continuous and dull with a heavy feeling. He reported the severity to be 8/10, on average. He described this to be a severe, burning, sharp pain, which could make him nauseous and fainting with any physical activity such as running, jumping, sexual intercourse, and physical examination. He also reported significant sensitivity and allodynia. Prior to our consultation, he was investigated with an ultrasound and CT scan on the right side. Since they showed some signs of edema and possible epididymitis, he was treated with antibiotics, without much improvement. He was also tried on nortriptyline 10 mg and (lyrica) pregabalin 150 mg BID, without much improvement. There were no other comorbidities or allergies. He was referred to us for the possibility of inguinal nerve blocks. On examination, he was anxious and quiet worried. His gait and posture were normal. His scrotal examination showed an empty scrotal sac on the left side and a highly sensitive inguinal region and scrotal sac on the right side. There were no signs of infection, swelling, or redness. There were no signs of inguinal or femoral hernia. The maximum tenderness was found to be just at the pubic tubercle and below, extending up to the whole of the right side of scrotum and also slightly over the medial side of thigh. Since the area of the lower abdomen and groin can be supplied by IL, IH, or GF nerve, we decided to perform separate diagnostic blocks to confirm the diagnosis and for a possible treatment. Initially, he underwent an ultrasound guided IL and IH nerve block, by the corresponding author, using 2 mL of 2% lidocaine and 2 mL of 0.25% bupivacaine mixed with 40 mg of depomedrol. The sensory block achieved did not cover the area of his pain. Approximately a month after that we performed an ultrasound guided GF nerve block using 2 mL of 2% lidocaine and 2 mL of 0.25% bupivacaine mixed with 40 mg of depomedrol.

### 2.1. Description of the Procedure

With patient in supine position, the inguinal area and the area above the femoral vessels were uncovered and wiped with chlorohexidine solution. A high frequency, linear, high resolution probe (GE Ultrasound, LOGIQ e machine) was initially kept perpendicular to the inguinal ligament just above the femoral vessels (Figure 1).

**Figure 2:**
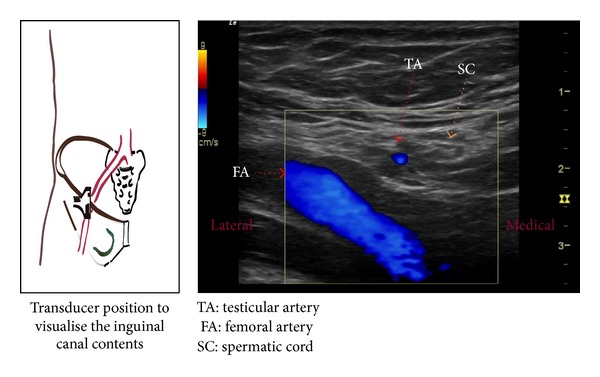
Visualization of structures in the inguinal canal and transducer orientation.

**Figure 3:**
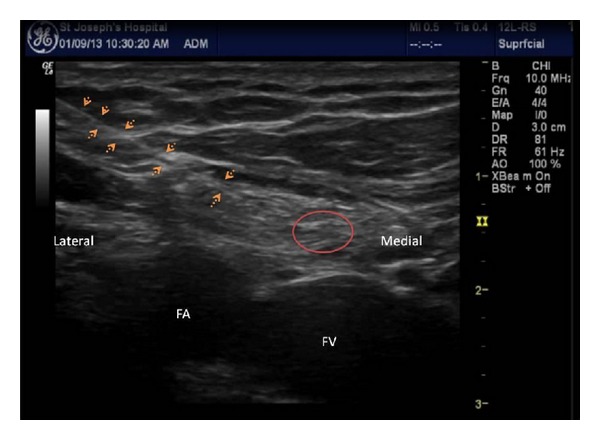
Injection into the inguinal canal to block the genitofemoral nerve.

A cephalad movement of the probe identified the iliac artery splitting into femoral and external iliac arteries. This corresponds to the level of the internal inguinal ring [6]. An oval structure lying medial and superficial to the femoral artery is the inguinal canal with its contents. A longitudinal view of the femoral artery is also identified at the same site (Figure 2). The contents of the inguinal canal were identified clearly, with testicular vessels shown laterally and spermatic cord shown medially. We used an in-plane approach to direct the needle towards the spermatic cord to block the genital branch of the GF nerve, using a 50 mm echostim needle (Benlan, Ontario, Canada) (Figure 3). Soon after the block, the patient noticed considerable improvement and tested it by jumping and running, to see if it hurts. The intensity of pain came down to 4/10, and the attacks of sharp pain became infrequent. The initial relief lasted 3 months, and he had a similar effect for the 2nd injection which lasted for 6 months. With an aim to prevent recurrence, he was tried on long acting tramadol 100 mg taken one a day. He underwent a 3rd injection after which his pain relief has continued beyond 12 months. He continues to be fully functional and is able to take part in normal physical activities.

## 3. Discussion 

Our report demonstrates that an ultrasound guided GF nerve block can be an effective treatment for genitofemoral neuralgia. However, it is critical to identify the involved nerve by performing diagnostic nerve blocks of the inguinal nerves, and the GF nerve to rule out their involvement. Similar to the IL nerve, the genitofemoral nerve arises from L1 and L2 (lumbar segments) and forms a part of the lumbar plexus. It predominantly carries sensory fibres, except the cremasteric motor fibres. The nerve lies on the surface of psoas major muscle, crosses the ureter on its descent, and divides into a genital and a femoral branch at a variable point above the inguinal ligament. The femoral component continues along the femoral sheath. The genital branch, also called the external spermatic nerve, gets into the inguinal canal and lies alongside the spermatic cord (round ligament in females). It carries sensory fibres from the lateral and posterior aspect of scrotum (mons pubis and labium majus in females) [7].

Most injuries to the GF nerve occur with hernia repair and pelvic surgeries such as urethral sling [8]. Other causes include blunt trauma, nephrectomy, appendicectomy, and ureterectomy [5]. It is difficult to comment on the exact nature of injury. Bischoff et al. performed a study to see whether intraoperative identification of these nerves, during hernia surgery, makes any difference. Out of 244 patients, GF nerve was identified in 21% of patients, compared to 94% and 97%, respectively, for IL and IH nerves. However intraoperative identification, and hence possibly a much safer technique, did not make a difference on the incidence of chronic pain [9]. The low identification rate is related to limited dissection, as recommended, near the course of GF nerve which may lie lateral to the external spermatic vein [10]. Laparoscopic hernia surgeries pose similar, if not higher risks. The repair involves placing tacks to secure the mesh from pubic symphysis to anterior superior iliac spine. For one such case, Rho et al. used a fluoroscopic guided injection to successfully treat the condition [11]. It was perhaps easier and safer to do it under ultrasound guidance, which was not as popular at that time. Parris et al. used a CT guided transpsoas approach and ablated the GF nerve, after what seemed to be a successful diagnostic block [12]. The needle was placed, after several attempts, by confirming with electrical stimulation. Unfortunately the patient continued to have persistent pain even after the procedure. Even in this instance an ultrasound guided procedure, near the deep inguinal ring, might have been easier, without the technical difficulties and exposure to radiation. The potential causes of neuropathic pain in these conditions may include inflammation, entrapment, neuroma formation, or deafferentation. Steroid mixed with local anesthetic (LA) solution can block ectopic impulses [13]. Other potential mechanisms include mechanical and anti-inflammatory effect. It must be noted that any such injection must be done preferably at the site of injury or proximal, to be effective. GF nerve is retroperitoneal until it enters the deep inguinal ring; hence the injury sustained is usually at this level or beyond, and not proximal. The ultrasound guided technique effectively allows for visualisation at this point and can be used for nerve block and pain control. Previously described blind techniques involved injecting 10 mLs of solution just lateral to the pubic tubercle [14]. This can be nonspecific and does not help in differentiating it from the inguinal nerve block. Other options to treatment include operative neurectomy, temporary or permanent neurolysis by using alcohol for phenol, or radiofrequency techniques [15]. Most are invasive and it must be borne in mind that patients can suffer from significant deafferentation pain in a very sensitive area, which itself could be quiet painful.

Through this report, we would like to highlight the need for a differential block, a safe and effective diagnosis, possibly leading to long duration of treatment. All these can be achieved using an ultrasound guided procedure.
